# Childhood exposure to danger increases Black youths’ alcohol consumption, accelerated aging, and cardiac risk as young adults: A test of the incubation hypothesis

**DOI:** 10.1017/S0954579425000264

**Published:** 2025-04-28

**Authors:** Steven R.H. Beach, Sierra E. Carter, Mei Ling Ong, Justin A. Lavner, Steven M. Kogan, Katherine B. Ehrlich, Man-Kit Lei, Ronald L. Simons, Olutosin Adesogan, Frederick X. Gibbons, Meg Gerrard, Robert A. Philibert

**Affiliations:** 1 Department of Psychology, University of Georgia, Athens, GA, USA; 2 Center for Family Research, University of Georgia, Athens, GA, USA; 3 Department of Psychology, Georgia State University, USA; 4 Human Development and Family Science, University of Georgia, Athens, GA, USA; 5 Department of Sociology, University of Georgia, Athens, GA, USA; 6 Department of Psychological Sciences, University of Connecticut, Storrs, CT, USA; 7 Department of Psychiatry, University of Iowa, Iowa City, IA, USA; 8 Behavioral Diagnostics, Coralville, IA, USA

**Keywords:** Danger, Inflammation, alcohol, DNAm-based aging, cardiac risk

## Abstract

Using the dual-pathway framework (Beach et al., 2022a), we tested a Neuro-immune Network (NIN) hypothesis: i.e., that chronically elevated inflammatory processes may have delayed (i.e., incubation) effects on young adult substance use, leading to negative health outcomes. In a sample of 449 participants in the Family and Community Health Study who were followed from age 10 to age 29, we examined a non-self-report index of young adult elevated alcohol consumption (EAC). By controlling self-reported substance use at the transition to adulthood, we were able to isolate a significant delayed (incubation) effect from childhood exposure to danger to EAC (β = −.157, p = .006), which contributed to significantly worse aging outomes. Indirect effects from danger to aging outcomes via EAC were: GrimAge (IE = .010, [.002, .024]), Cardiac Risk (IE = −.004, [−.011, −.001]), DunedinPACE (IE = .002, [.000, .008]). In exploratory analyses we examined potential sex differences in effects, showing slightly stronger incubation effects for men and slightly stronger effects of EAC on aging outcomes for women. Results support the NIN hypothesis that incubation of immune pathway effects contributes to elevated alcohol consumption in young adulthood, resulting in accelerated aging and elevated cardiac risk outcomes via health behavior.

## Introduction

Black youth growing up in the United States disproportionately confront challenges linked to dangerous environments (Keppel et al., 2002; McArdle et al., 2007) and exposure to discrimination (Hurd et al., 2014; Stock et al., 2017). Furthermore, disparities embedded in social, legal, and political manifestations of structural racism affect multiple contexts of development for Black youth (Zapolski et al., 2014). Disparities in stressful early experiences are associated with long-term consequences for health in adulthood (Gunnar & Quevedo, 2007; Lupien et al., 2009) in general, and for Black Americans’ health outcomes in particular (Williams, 2012). Downstream effects of these disparities include increased likelihood of sudden cardiac arrest (Carnethon et al., 2017), early onset of blood pressure problems, and risk for multimorbidity (Lim et al., 2018; Thorpe et al., 2016). Characterizing risk pathways that link early adverse exposures to adult health outcomes is critical for identifying opportunities for preventive intervention.

Empirical evidence with Black American samples suggests that adverse events in childhood can “get under the skin” to influence long-term health outcomes via both behavioral and immune pathways (Beach et al., 2022b; Cole, 2014; Lei et al., 2020). The “dual pathway” model specifies two independent mechanisms through which behavioral and immune mechanism affect health (Beach et al., 2022a). Recent theorizing, however, suggests that transactions between behavioral and immunological pathways may result in the *incubation* of health-related vulnerabilities. Incubation refers to a process whereby effects of an early-life experience—especially a stressful or traumatic one—do not manifest right away, but rather emerge later in life in a way that may not seem obviously connected to the original event. The Neuro-immune Network (NIN) model (Nusslock & Miller, 2016) proposes that early experiences of stress and adversity work in this manner, often producing changes in peripheral biological systems that create chronic inflammation and, in turn, influence the development of several central processing systems by modifying several key control systems related to threat, reward, and executive control. These changes are expected to combine with normal developmental changes in family-based resilience, work demands, and chronic stressors, to increase vulnerability to substance use in young adulthood, including the development of problematic alcohol use as a way of self-medicating. It is expected that these problems may emerge among those who did not show substance use problems earlier in life (Nusslock & Miller, 2016), suggesting a vulnerability that goes beyond other developmental contributions to alcohol and substance use problems.

A number of studies support the hypothesis that early adversity results in elevated inflammatory biomarkers well into adulthood (Danese et al., 2007, 2009; Kiecolt-Glaser et al., 2011). In the neuroimmune network (NIN) model, Nusslock and Miller (2016) hypothesized that this lasting effect of early adversity on inflammation would affect several health behaviors, including substance misuse, at a delay across the lifespan. Specifically, early adversity was hypothesized to sensitize the cortico-amygdala regions of the brain and the immune cells that propagate inflammation (i.e., monocytes and macrophages), programming them to mount exaggerated responses to infections and injuries. As this low-grade inflammation spread to the brain, it was expected to further accentuate threat-related processes in the cortico-amygdala circuit, attenuate reward-related processes in the cortico-basal ganglia circuit, and dampen executive control-related processes linked to regions of the prefrontal cortex. As described in greater detail by Nusslock and Miller (2016), the amplified bidirectional pathways linking peripheral inflammation with neural circuitries were expected to give rise to self-medicating behaviors (see Nusslock & Miller, 2016, p. 24).

A parallel line of theorizing by Cole (2014) places lasting immune effects in evolutionary context, identifying a broad “conserved transcriptional response to early adversity” (CTRA). Specifically, the Cole team note that the immune system comprises two rather distinct programs: proinflammatory cytokine genes that combat tissue damage, bacteria, and other extracellular pathogens, and antiviral genes that produce antibodies and target intracellular pathogens such as viruses. They argue that adversity (threat or danger) leads to increased expression of the inflammatory program, coupled with decreased expression of the antiviral program, as the organism prepares for possible attack and injury. Cole (2014) labels this pattern of gene expression the conserved transcriptional response to adversity (CTRA). The CTRA comprises a shift toward greater anti-microbial activity, and associated blood cell-types, as well as reduced anti-viral activity, and associated cell-types. This approach provides alternative, parallel, methods of assessment to capture the hypothesized pro-inflammatory shift in response to early adversity. As described by Simons et al. (2017) using the ratio of pro-inflammatory to antiviral cell types (ITACT Ratio), it is possible to create an index of pro-inflammatory changes in response to adversity using the ratio of cell-types that is well-grounded in evolutionary theory (e.g. Cole, 2014).

From the NIN model perspective, substance use and health vulnerabilities may be incubating due to increased inflammatory processes even in young people with low rates of risk behavior during adolescence. The incubation concept also helps explain how early exposure to poverty and violent crime, which is more common for Black youth than for White youth (e.g., Pinchak et al., 2022), can contribute to overall propensity for alcohol problems in adulthood even though Black youth are not at elevated risk for early onset of alcohol consumption relative to White youth (Zapolski, 2014). Importantly, there is substantially greater exposure of Black youth than White youth to community violence, including among pre-adolescents (Fitzpatrick & Boldizar, 1993; Gladstein et al., 1992; Selner-O’Hagan et al., 1998). For example, combining across several categories of community violence including witnessing a beating, robbery, stabbing, shooting, or murder, Campbell and Schwarz (1996) found quite elevated levels of exposure among urban youth, and Gladstein et al. (1992) reported that substantial numbers of inner-city youth in Maryland had been exposed to severe violence and knew someone who had been victimized personally. Accordingly, many Black youth, including pre-adolescents, are both aware of, and affected by, community violence. Below we summarize the dual pathway model to clarify the key pathways relevant to the “incubation” hypothesis.

### The behavioral pathway

Among Black youth, early exposure to discrimination has been linked prospectively to early onset substance use across a number of contexts (Belsky et al., 1991; Figueredo et al., 2005; Gibbons et al., 2012; Hurd et al., 2014; Metzger et al, 2017, 2018). Informed by the dual pathway model, Beach et al. (2022a) investigated accelerated aging as a potential outcome resulting from early exposures to discrimination via early onset substance use. Prospective analysis suggested that early onset substance use promoted accelerated aging based on metrics focused on health and mortality indicators [e.g., GrimAge, PhenoAge, and Telomere length (see Levine et al., 2018; Lu et al., 2019a, 2019b), but not measures of accelerated DNA-methylation aging focused on chronological age (Hannum et al., 2013; Horvath, 2013)]. This patterns suggests that indices such as accelerated GrimAge and DunedinPACE might be more sensitive to the health-related impact of childhood exposures and risky substance use behavior (see also McCrory et al., 2022).

### The immune pathway

In a separate literature, developmental exposure to danger has been proposed to elicit epigenetic and brain-related changes (McLaughlin et al., 2014). Although childhood exposure variables are often highly intercorrelated, making it difficult to tease apart independent effects of different facets of difficult childhood environments, basic research on neurodevelopment is consistent with the NIN hypothesis that exposure to danger may have distinct effects on altered immune responses (e.g., Lam et al., 2022) and brain circuitry (e.g., McLaughlin et al., 2014). Lam et al. (2022) found that youth-reported exposure to violence and neighborhood-level murder rate was associated with more pronounced pro-inflammatory cytokine production. McLaughlin et al. (2014) found that cues signaling dangerousness in the early environment elicited epigenetic changes that regulated Hypothalamic/pituitary/adrenal axis activity, hypotheses that were further developed by Kremer et al. (2020), who showed effects of exposure to dangerous environments in childhood on methylation of FKBP5. The NIN hypothesis (Nusslock & Miller, 2016) proposed that early adversity has the potential to amplify crosstalk between peripheral inflammation and neural circuitries subserving threat-related processes, influencing development of brain circuitries across development. As a consequence, early exposure to dangerous environments is thought to shape reactivity to stressors, altering cellular environments and producing epigenetic reprogramming across peripheral tissues like blood as well as the central nervous system.

One focus of attention with regard to epigenetic reprogramming of tissues in response to exposure to dangerous contexts in childhood are changes in “methylation” of *FKBP5* (Kremer et al., 2020). To review, methylation of a regulatory motif occurs when a methyl group is attached to a segment of deoxyribonucleic acid (DNA) at a CpG site (i.e., a region of DNA with cytosine followed by guanine linked by a phosphate group). Greater methylation, particularly in loci close to transcription start sites, is typically associated with greater inhibition of gene expression. Conversely, demethylation is typically associated with increased gene expression.

Levels of *FKBP5* expression are known to be associated with regulation of inflammatory activity (e.g., Erlejman et al., 2014; Maiarù et al., 2016). Further, there are two CpGs of particular interest on *FKBP5* identified by Zannas and colleagues (cg20813374 and cg00130530) (Zannas et al., 2019). These loci are in close physical proximity to each other and to the *FKBP5* transcription start site (i.e., less than 500 bp in each case), and also are correlated with each other. Early exposure to danger predicts demethylation of *FKBP5*, which, in turn, predicts greater pro-inflammatory propensities and accelerated aging (Beach et al., 2022a).

### Adding “Incubation” of Immune Pathway Effects on Behavior to the Dual Pathway Model

We previously posited and found independent effects on accelerated aging attributable to the behavioral and immune pathways (Beach et al., 2022a). Specifically, early substance use and other early risky behaviors were not correlated with FKBP5–2 demethylation, and had independent associations with accelerated aging. Left unexamined, however, were potential links between FKBP5–2 demethylation and substance use that may become apparent only later in young adulthood, such as those predicted by the NIN framework to result from incubation. Accordingly, there was no test of incubation (i.e., delayed) effects (Miller & Raison, 2016; Nusslock & Miller, 2016; Schiller et al., 2021). An association that becomes apparent in young adulthood, and goes beyond what would be expected based on early onset substance use, would be consistent with increased vulnerability to substance use in young adulthood due to the immune pathway. Supporting this expectation, other research has found that early adversity is associated with chronic inflammation and can influence reward response systems (Miller et al., 2024). Likewise, one might also anticipate delayed (incubation) effects because of the differing time scales for development of immune and nervous system effects (Lee et al., 2014; Leppänen & Nelson, 2009), or because of insufficient variability in level of substance use at younger ages.

**Figure 1.**
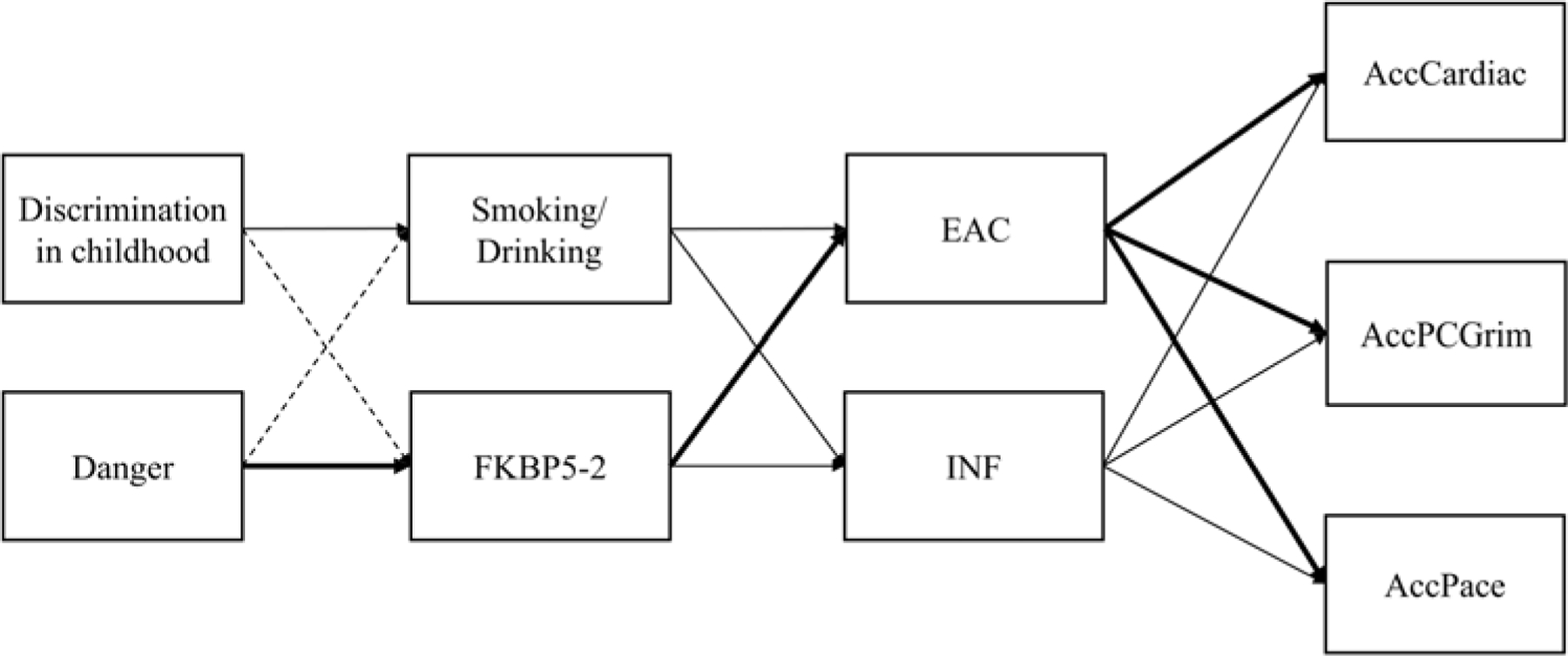
Dual pathway model with Incubation highlighting delayed effects from the immune pathway to elevated alcohol use in young adulthood beyond effects attributable to the behavioral pathway and resulting in significant indirect pathways from early childhood exposures to health outcomes in young adulthood (Bolded) beyond those attributable to the model without hypothesized incubation effects. Note: Danger = Exposure to dangerous environments in childhood; EAC = composite Non-self-report elevated alcohol consumption; INF = inflammatory to anti-viral cell-type-ratio.

The resulting dual-pathway model with incubation-related effects included is presented in Figure 1.

As shown in Figure 1, the dual pathway model (expanded to include EAC and incubation) includes the behavioral pathway beginning with early exposure to racial discrimination which contributes to risky health behavior (Gibbons et al., 2004; Zapolski et al., 2016) and ultimately health outcomes (Carter et al., 2019; Geronimus et al., 2010; Zapolski et al., 2014). It also includes the immune pathway beginning with exposure to danger in childhood which leads to downstream consequences for regulation of *FKBP5*, inflammatory tendencies, and ultimately health outcomes. The revised model shown in Figure 1 now adds the prediction of a significant delayed association, resulting in an “incubation” pathway (bolded in Figure 1) from exposure to danger in childhood to regulation of *FKBP5* (demethylation) and from there to elevated alcohol consumption (EAC) in young adulthood, beyond what would be expected based on earlier risky substance use. This revised model indicates that delayed (incubation) effects will contribute to elevated alcohol consumption and ultimately health outcomes beyond the effect of continuity in alcohol use from early onset of drinking and cigarette smoking at the transition to adulthood. Examining this model allows us to test both the significance of the pathway from *FKBP5* to elevated alcohol consumption later in adulthood (net of early substance use) as well as to examine the significance of indirect effects from early exposure to danger to health outcomes via incubation effects.

Whereas the initial test of the dual pathway model (Beach et al., 2022a) relied on self-reported substance use during the transition to adulthood (age 18–23) to characterize the trajectory of the behavioral pathway, the current analyses added a composite of three non-self-report indicators of heavy alcohol consumption later in young adulthood (age 29) to gauge potential delayed emergence of associations between the immune pathway and substance use later in young adulthood. We also added a recently developed measure of cardiac risk as an additional health-outcome to supplement our previous focus on DNAm-based measures of accelerated aging. As we did previously, we use data from the Family and Community Health Study (FACHS) to examine prospective reports of stressors experienced when participants were 10 years old and examine potential indirect pathways to later health-related indices in young adulthood (age 29 years). Below we discuss several methodological issues in more detail to provide additional context for our measurement decisions and to explicate the model.

### Methodological considerations

#### Measuring young adult alcohol consumption

First, there are several reasons to expect that *self-reported* level of substance use, including smoking and drinking, do not always accurately reflect actual level of consumption (e.g., Andersen et al., 2017; Beach et al., 2022b). Alcohol use appears to be substantially underreported in young adulthood relative to reports at the transition to adulthood in the current sample. We found that reliable and independent non-self-report indicators of alcohol use in young adulthood are related to each other, and with self-reported alcohol use at the transition to adulthood (ages 19–23), but not with self-reported alcohol use in young adulthood (age 29) (Beach et al., 2022b). Further, self-reported substance use is typically not associated with DNAm indices of accelerated aging (Ryan et al., 2020), suggesting more general measurement issues that may limit the utility of self-reported alcohol use in predicting health outcomes. Of particular concern in the context of predicting incubation effects is that reports of low or moderate drinking may be more accurately reported than is heavy drinking (Northcote & Livingston, 2011), and accuracy may decline further with age due to stigma (Andersen et al., 2017), both of which would tend to obscure incubation effects.

Given these considerations, in the current study we use three independent, *non*-*self-report* indices of elevated alcohol consumption (EAC) using blood-based assessments to create a composite index. As described in greater detail in the Method section, the three indices are different in their focus and methods. We first used a reference-free, droplet digital PCR assessment, the Alcohol T-Score (ATS; Dawes et al., 2021; Miller et al., 2021), a method that does not use the array-based methylation data used for outcomes in the current investigation. In addition, the ATS was designed to distinguish heavy alcohol users receiving inpatient treatment from community controls. Second, we utilize carbohydrate-deficient transferrin (CDT) assessments, a generally accepted biomarker of heavy alcohol consumption that uses changes in sialic acid glycoconjugation of transferrin, to provide a window on heavy alcohol intake during the previous three-weeks (Bortolotti et al., 2018). The CDT assesses alcohol-related changes in liver function and so captures health-related impact of heavy alcohol use. As our third non-self-report index of EAC, we used the Methyl DetectR (MDR) index developed by Hillary and associates (Hillary & Marioni, 2020), which utilized a machine learning approach to extract a heavy drinking signature from genome wide methylation data provided by 4450 participants in the Generation Scotland Study (Smith et al., 2006). The instrument was designed to predict self-reported average weekly alcohol consumption. As such, it captures sustained elevated alcohol consumption but not necessarily binge or problem drinking in the way that the ATS and CDT do. In addition, there was no effort to link problematic levels of use with the MDR. Accordingly, the three methods we utilize to construct a composite index of EAC were developed independently and provide different windows on EAC.

#### Gender differences

A second methodological consideration, as noted by Chen & Jacobson (2012), is that there are several reasons to expect gender-related differences in patterns of development of substance use across adolescence and early adulthood. First, although substance use often increases during middle/late adolescent years among both Black and White adolescents and peaks around early or middle 20s, young women tend to have higher levels of substance use in early adolescence than do young men, with males often catching up and surpassing them in early adulthood and White youth show greater increases from ages 9 to 16, with increases for Black youth occurring later (Duncan et al., 2006; Jackson et al., 2002). Second, there are gender differences in peer selection processes that may affect maintenance and level of substance use in early adulthood (Windle & Windle, 2018). Finally, it has been observed that young Black men are more likely than young Black women to show increasing substance use across the age span from 17 to 29 (Chen et al., 2018). Accordingly, there may be greater opportunities for elevated alcohol consumption to show effects on health for young Black men. However, given lack of prior findings examining this issue, we treat analyses examining gender differences in incubation effects and in alcohol effects on health as exploratory.

#### Measuring young adult accelerated aging

It is also important to consider how accelerated aging is measured. As noted earlier, in prior research we showed that the effect of substance use on accelerated aging was not well-captured by earlier DNA-based methylation measures of accelerated aging trained on chronological age (Beach et al., 2022a), but did show associations with more recently developed DNA-based methylation measures of accelerated aging that had been trained on health-related or mortality data. Accordingly, we focus on two of the more recently developed accelerated aging measures. First, we used the PCGrim version of GrimAge (Lu et al., 2019a) which was trained to predict time to death due to all-cause mortality. Second, we used the DunedinPACE measure (DunedinPACE; Belsky et al., 2022) which was trained to assess change across 19 organ systems to capture speed of overall aging. For continuity with prior work, in supplementary sensitivity analyses we examine effects of the revised dual-pathway model using the two other health-related DNAm-aging outcomes previously examined in Beach et al. (2022a) (i.e., PhenoAge and Telomere length).

#### Measuring Cardiac Risk

EAC is also thought to be a contributing cause for various forms of cardiometabolic risk (Harris, 2023), exerting its effect through multiple pathways (Mathews et al., 2015). For example, those with an alcohol use disorder have twice the rate of coronary heart disease as those with average alcohol consumption (Roerecke & Rehm, 2014). Given the association of alcohol consumption with increased cardiometabolic risk, we expanded our investigation to include a measure of elevated cardiac risk based on risk indicators identified by Cardio Diagnostics. This approach uses a suite of six methylation loci to identify increased risk of an incident cardiac event (cg03725309, cg12586707, cg04988978, cg17901584, cg21161138 and cg12655112) (Philibert et al., 2023a, 2023b). Lower methylation levels at each of these six loci are associated with increased cardiac risk. Although the clinical method uses methylation sensitive digital PCR to determine status, level at each locus can also be obtained using array data as we do in the current study. In prior research we found that demethylation of FKBP5–2 was associated with increased cardiometabolic risk using the Framingham heart age measure (Beach et al., 2021), suggesting the potential for the dual pathway model to predict cardiac risk as well. In exploratory analyses (provided in Supplementary Figures S1 and S2) we also examined prediction of diabetes and elevated HbA1c.

#### Specific hypotheses to be tested

Collectively, we tested several hypotheses in the current study:

1. Guided by the NIN model, we hypothesized that a significant correlation would emerge in young adulthood between FKBP5–2, marking the inflammation pathway, and our composite, non-self-report index of elevated alcohol consumption (EAC). We also hypothesized that there would be an association between substance use at the transition to adulthood and young adult EAC. Based on prior results, we expected no association between FKBP5–2 and earlier smoking and drinking at the transition to adulthood.
2. We hypothesized that using multivariate regression analyses, the association between FKBP5–2 and EAC would continue to be significant after accounting for the effect of earlier reported substance use, age, and gender, and would show a significant association of FKBP5–2 with EAC beyond the effect of the behavioral pathway and control variables. We also explored the possibility that the impact of FKBP5–2 on EAC would be greater for males than for females and this would be reflected in a significant interaction effect of gender with FKBP5–2 in the prediction of EAC.
3. We hypothesized that there would be significant indirect effects from early exposure to danger to accelerated aging and increased cardiac risk in adulthood via the delayed (incubation) effect pathway through EAC, net of the behavioral pathway and the inflammation pathway. Health indices were PCGrim, DunedinPACE, and cardiac risk.

We explored sensitivity of the model in two ways. First, we examined sensitivity to additional predictors by re-introducing all predictors and mediators from the original model (Beach et al., 2022a). Second, we examined sensitivity to outcomes by exploring effects on the other health-related DNAm-based indices of accelerated aging used in the original model (i.e. PhenoAge and Telemere Age) as well as type-II diabetes and elevated HbA1c. Finally, we explored the potential for gender-related differences in the impact of EAC on health-related outcomes.

## Method

### Participants

Hypotheses were examined using data from a longitudinal study of 889 Black American families with a fifth-grade child that was initiated in 1997 in Iowa and Georgia, i.e., the FACHS sample. Youth mean age at baseline (W1) was 10.56 years (*SD* = .63; range 9–13). The average family per capita income reported by children’s primary caregivers at baseline was $6,956, with 36% of the families below the poverty line and 51% of the respondents self-identifying as single parents. Data used in the current study were also collected in 2005–2007 (W4), 2008–2009 (W5), and 2010–2011 (W6), when the participants in the current study were, on average, aged 18.7, 21.5, and 23.5 years, respectively. Data were also collected in 2015–2016 (W7), when the participants in the current study were, on average, age 28.7 years (*SD* = .79; range: 27–31). In the 2015–2016 (W7) data collection we included blood draws, allowing analysis of cell-type variation; methylation-based indices of diet, alcohol use, and smoking; assessment of THC to determine marijuana use; as well as creation of DNA-methylation-based indices of aging. Because of limitations related to the collection of blood, in the 2015–2016 data collection, we only included participants still residing in Georgia, Iowa, or a contiguous state who could be visited at home by phlebotomists. Along with attrition due to death, incarceration, or being unreachable, this resulted in a potential participant pool of 556 individuals, 470 of whom (182 men and 288 women) provided blood at age 29. Of these, 449 (96%) were successfully assayed and comprise the sample for the current analyses (172 men and 277 women). We examined potential differences between those included in the final sample and those lost to attrition on baseline predictors (danger and discrimination) as well as demographics (age and sex) (see Supplementary Table S1). There were no differences in baseline predictors. Both age and sex, which did show an association with attrition, are controlled in all analyses.

### Procedure

All study protocols and procedures for age 29 participants were approved by the Institutional Review Board at the University of Georgia (Title: FACHS weathering – Protocol study number 00006152). All adult participants provided informed consent and youth provided assent. Black university students and community members collected data after receiving training in the administration of the interview to increase validity and enhance rapport and cultural understanding. Interviews at baseline were administered in respondents’ homes and took on average about 2 to 2 ½ hours. In 2015–2016, data collection included blood draws, providing the basis for non-self-report indices. Blood was shipped via courier to a laboratory at the University of Iowa for processing. Data and analysis code are available upon request from the first author. The analyses were not preregistered.

#### Assessment of methylation.

Whole blood samples were collected in sodium citrate tubes and were processed into DNA using cold protein precipitation. The resulting DNA was stored at −20° C until usage. DNA methylation-based assessments were conducted with the Infinium (Illumina, San Diego, CA, USA) HumanMethylationEPIC 850 BeadChip. This array contains 865,918 probes recognizing CpG positions of known transcripts, potential transcripts or CpG islands. Participant DNA samples were randomly assigned to “slides/chips” that were bisulfite converted in 96 well batches. A replicated sample of DNA was included in each plate to aid in assessment of batch variation and to ensure correct handling of specimens. The replicate samples were examined for average correlation of beta values between plates and was found to be greater than 0.99. Prior to normalization, DNA methylation data were filtered based on the following criteria: (a) samples were examined to identify any “poor quality samples” containing 1% or more of CpG sites with detection *p* < 0.05 (no samples failed this criterion), (b) sites were removed if a bead count of <3 was present in 5% of samples, and (c) sites with a detection *p* < 0.05 in 1% of samples were removed.

The beta value at each CpG locus was calculated as the ratio of the intensity of the methylated probe to the sum of intensities of the methylated and unmethylated probes. Quantile normalization methods were used, with separate normalization of Type I and Type II assays, as this approach has been found to produce marked improvement for the Illumina array in detection of relations by correcting distributional problems inherent in the manufacturers’ default method for calculating the beta value. In addition, values were background corrected using the “noob” method (Triche et al., 2013). Finally, as described below, beta values after quantile normalization were used to calculate DNA methylation-based aging indices for each participant. Methylation values were also used to calculate cell-types for our measure of inflammation. In addition, methylation values were used to provide an index of health behavior.

### Measures

#### Prospective predictors

##### Childhood Exposure to Danger.

Three items were used to capture the child’s report of exposure to community dangerousness at W1 when they were approximately 10 years old. The three items asked about the following experiences over the past six months: (1) a fight in your neighborhood in which a weapon like a gun or knife was used; (2) a robbery or mugging; and (3) a sexual assault or rape. All indicators were coded on a scale from 1 = *never* to 3 = *often*. The composite index of exposure to danger was created by taking the mean of the three indicators. Scores ranged from 1 to 3 (M = 1.40, SD = .50, Alpha = .614). Prior analyses indicated that exposure to neighborhood danger was distinct from other dimensions of childhood adversity such as family SES, parenting, and discrimination (Beach et al., 2022a), and was uniquely related to FKBP5–2.

##### Discrimination.

Participants completed the Schedule of Racist Events (Landrine & Klonoff, 1996) (13-item modified version) at Wave 1. The measure asks participants how often they have experienced discriminatory events, e.g., “How often has someone said something insulting to you just because you are African American?”; “How often have you been treated unfairly because you are African American instead of White?” rated from 1 = *never* to 4 = *frequently*). The scale was modified to make it more appropriate for non-adult respondents. The revision simplified some language, and items on workplace discrimination were replaced with items about general experiences in the community. Cronbach’s alpha was .859.

#### Mediators of early exposure effects

##### FKBP5–2.

To provide an index of methylation level for the two methylation sites on FKBP5 previously shown to be related to childhood exposures (Beach et al., 2022a), we examined level of methylation at cg20813374 and cg00130530 at W7 when participants were, on average 29 years old. The two CpG sites were correlated *r* = .416, *p* < .001, supporting previous work indicating that they co-vary and could be combined into a meaningful index. To create a single index, the quantile-normalized beta values at each CpG site were averaged, resulting in a single methylation index of impact of early exposure to danger on FKBP5 methylation.

##### Substance use (smoking/drinking).

Early substance use-related behaviors were operationalized as self-reported smoking and binge alcohol use at Waves 4, 5, and 6 when participants were, on average, 18.7, 21.5, and 23.5 years old. Participants were asked one question at each wave for heavy alcohol consumption (During the past 12 months, how often have you had a lot to drink, that is 3 or more drinks at one time?) and cigarette use (How many cigarettes have you smoked in the last 3 months?). Items were combined within wave by taking the mean of the standardized scores for each item, and then averaged across waves. The alpha for the scale was .778.

#### hypothesized downstream mediators

##### Elevated Alcohol Consumption (EAC).

We created a composite non-self-report index of EAC by normalizing each of three *non-self-report* indices of EAC and averaging them. The three components of the EAC composite were as follows: 1) *Carbohydrate-deficient Transferrin*. Carbohydrate-deficient transferrin (CDT) is a type of molecule deficient in carbohydrate sialic acid that carries iron into the bloodstream. Because alcohol consumption inhibits sialic acid from attaching to transferrin (Stibler, 1991), the CDT is generally considered a good *non-self-report index* for alcohol testing with sensitivity and specificity of approximately 70% (Bortolotti et al., 2018). CDT is highly sensitive to heavy alcohol consumption (i.e., more than 5–7 standard drinks per day), and so can be used to assess excessive drinking (Hock et al., 2005). CDT remains elevated for up to 3 weeks. 2) *Alcohol DetectR.* Hillary and associates (Hillary & Marioni, 2020) developed the Alcohol DetectR (MDR) using a machine learning approach and data from 4450 participants in the Generation Scotland Study (Hillary & Marioni, 2020; Smith et al., 2006) to predict self-reported weekly alcohol consumption. Accordingly, scores reflect a range of usual consumption levels, but not episodic or binge use. Methyl DetectR values for alcohol consumption per week were calculated using the code supplied by the University of Edinburgh website. https://wellcomeopenresearch.org/articles/5-283. 3) *Alcohol T Scores (ATS)* use methylation sensitive droplet digital PCR MSddPCR assays to characterize methylation levels at four loci to provide a metric predictive of EAC (Philibert et al., 2019). This tool has an AUC of 0.96 for distinguishing community participants from those admitted for treatment of EAC (Bortolotti et al., 2018; Miller et al., 2021) and provides objective assessments of substance use without concerns about effects of probe normalization or DNA quality on accuracy of methylation status (Pinheiro & Emslie, 2018). Methylation status at the four loci (cg02583484, cg04987734, cg09935388 and cg04583842) in the ATS panel was assessed using primer probe sets from Behavioral Diagnostics (Coralville, IA) and both droplet digital PCR reagents and equipment from Bio-Rad (Hercules, CA) according to our previously described protocols (Philibert et al., 2020). Increasing values are predictive of increasing alcohol consumption. The mean ATS score in the current sample was .778, with 28.1% scoring in the elevated range (> 2.35).

The three non-self-report indicators of EAC were correlated with each other (*r*s ranging from .369 to .407). All were also significantly correlated with self-reported smoking at Waves 5 and 6 as well as self-reported alcohol use at Waves 5 and 6. See Supplementary Table S2.

##### Inflammatory/Antiviral Cell Type Ratio (INF).

To index mobilization of hemopoietic stem cells, resulting in a pro-inflammatory shift toward production of monocytes and natural killer cells relative to T and B cells (Cole et al., 2011; McKim et al., 2018), we examined an index of cell types using the blood drawn at W7 (age approximately 29). We followed the procedure developed by Houseman et al. (2012), and used the formula proposed by Simons et al. (2017). Specifically, the “EstimateCellCounts” function in the minfi Bioconductor package was performed to assess individual differences in the distribution of cell types. The peripheral white blood cell contribution was subclassified into five different cell types. Two of these cell types—monocytes and natural killer cells— are associated with the innate immune system and an inflammatory response. The other three cell types—CD4+ T cells, CD8+ T cells, and B cells—are associated with the adaptive immune system and antiviral processes. The following equation was used to calculate the prevalence of inflammatory relative to antiviral cell types:

$$\frac{\left(Monocytes+NaturalKiller\right)}{\left({CD4}^{+}T+{CD8}^{+}T+Bcells\right)}$$

Using this ratio, higher scores indicated increased dominance of the innate/inflammatory response. The correlation between the numerator (innate/inflammatory cells) and denominator (adaptive/antiviral cells) was *r* = −.146, *p* = .002. The mean ratio score was .261 (*SD* = .319) and the range was 0.00 to 5.29.

#### Health-related outcomes in adulthood

##### AccPCGrim.

To characterize effects on young adult health we used a DNAm-based index of accelerated aging and all-cause mortality developed by Lu et al. (2019a). This measure predicts time to death due to all-cause mortality (Lu et al., 2019a), and so provides a mortality risk estimate called “DNAm GrimAge.” GrimAge has demonstrated good predictive ability for time-to-death, time-to-coronary heart disease, time-to-cancer, and has also shown an association with computed tomography data for fatty liver/excess visceral fat, and age at menopause (Lu et al., 2019a). The Grim index includes 1030 CpG markers to forecast all-cause mortality. In the current investigation we utilized a recent methodological enhancement focused on the principal component of the Grim index, known as ‘PCGrim clock’ (Higgins-Chen et al., 2022). This version of the index has demonstrated improved technical reliability over the original versions. PCGrimAge was analyzed using the online “run_calcPCClocks_Accel.R program” found at PC-Clocks GitHub repository (https://github.com/MorganLevineLab/PC-Clocks/blob/main/CITATION.cff). In each case we used the Advanced Analysis option and the normalize data option. PCGrimAge was regressed on chronological age to yield the dependent variable of accelerated PCGrim (AccPCGrim).

##### AccPACE.

The DunedinPACE was introduced (Belsky et al., 2022) as an index of DNA methylation-based aging using 19 indicators of organ-system integrity across four time points spanning two decades, and summarizes change in EpiAge using a single-time-point DNA-methylation blood-test. Scores were also calculated using the code supplied by the developers at https://github.com/danbelsky. DunedinPACE includes 173 CpG sites. Values greater than 1 indicate accelerated aging. For consistency, DunedinPACE was regressed on chronological age to yield the dependent varable of accelerated DunedinPACE (AccPACE).

##### AccCardiac.

The Cardiac risk index used genome wide methylation data. After processing, *M* values for methylation level at each of the six coronary heart disease related sites (cg03725309, cg12586707, cg04988978, cg17901584, cg21161138 and cg12655112) used in our analyses were extracted and converted to beta values for use in our analyses. Prior research has validated each of these loci as indicators of coronary heart disease (Philibert et al., 2023a, 2023b), with demethylation indicative of increased risk.

Chronological age was controlled by regressing Cardiac Risk on chronological age so that scores reflect accelerated cardiac risk (AccCardiac).

### Plan of analysis

After computing simple correlations and descriptive statistics, we used multiple regression analyses to provide a focused test of the incubation hypothesis. We also used moderated regression to test the exploratory hypothesis of stronger incubation effects for men than women. To examine the full hypothesized model and examine indirect pathways to health outcomes, we used M*plus* 8 (Muthén & Muthén, 2017) to conduct path analyses. To assess goodness-of-fit, chi-squared statistics, the comparative fit index (CFI > .90 = acceptable; > .95 = good), Steiger’s root mean square error of approximation (RMSEA < .10 = acceptable; < .06 = good), and standardized root mean square error (Ximénez et al., 2022) (SRMR < .10 = acceptable; < .06 = good) were used to assess the significance of indirect effects, the 95% confidence interval (CI) was estimated using bias-corrected and accelerated bootstrapping. In line with recommendations by Yzerbyt et al. (2018), we report that all individual component coefficients in the pathway are significant as well as reporting significant indirect effects using bootstrapping with 30,000 resamples.

In sensitivity analyses regarding the influence of additional predictors, we examined the impact of including all variables used by Beach et al. (2022a). We also conducted sensitivity analyses using additional DNAm accelerated aging outcomes examined previously by Beach et al. (2022a) and using Type-II diabetes and HbA1c as outcomes.

Finally, we explored gender differences in the magnitude of the effects of EAC on health-related outcomes by introducing interaction terms for the pathways from EAC to aging outcomes.

## Results

### General description of associations

As shown in Table 1, there were significant correlations in the expected directions between early exposures and hypothesized mediators (e.g., danger with FKBP5–2 demethylation, *r* = −.117, *p* = .013; discrimination with substance use during the transition to adulthood, *r* = .167, *p* < .001). Also, exposure to danger and discrimination were correlated, *r* = .303, *p* < .001. Likewise, there were significant correlations in the expected direction between hypothesized mediators and outcomes. EAC correlated with AccPCGrim, AccPACE, and AccCardiac, with |*r*|s between .132 and .657; and INF correlated with AccPCGrim, AccPACE, and AccCardiac, with |*r*|s between .189 and .406. Consistent with the expectation of continuity in the behavioral pathway, there was a significant association between self-reported substance use at the transition to adulthood, i.e., smoking/drinking, and the composite index of non-self-report indicators of elevated alcohol consumption at age 29, i.e., EAC (*r* = .335, *p* < .001). However, there was not a significant association of early exposure to danger and either substance use at the transition to adulthood (*r* = .023, NS) or EAC *(r* = −.002, NS). These associations set the stage for the proposed tests of hypotheses regarding incubation effects linking early exposure to danger to EAC and through EAC to outcomes.

### Hypothesis 1: examination of simple associations

As hypothesized, the association between the inflammatory pathway and the behavioral pathway was not reflected in correlations with smoking/drinking at the transition to adulthood but was apparent in young adulthood (age 29). Specifically, we found that the FKBP5–2 regulatory motif was not significantly associated with self-reported substance use during the transition to adulthood (*r* = −.08, *p* = .092) but was associated with EAC later in young adulthood in the expected direction (*r* = −.203, *p* < .001).

### Hypothesis 2: examination of Incubation

The association of FKBP5–2 with EAC in young adulthood remained significant after accounting for the significant association of self-reported smoking/drinking during the transition to adulthood with EAC, and after accounting for age and gender. Using regression analyses, as shown in Table 2, column 2, controlling for age and gender, we found a significant increase in R^2^ (Δ *R*^2^ = .023, *p* = .001) by adding FKBP5–2 to smoking/drinking and demographic predictors (*β* = −.155, *p* = .001). In an additional exploratory analysis we examined the interaction of gender and FKBP5–2, showing modestly stronger effects of for men and women. Specifically, controlling effects of smoking/drinking at the transition to adulthood as well as gender, we examined the interaction of FKBP5–2 with gender. As can be seen in Table 2, column 3, when we examined the interaction of FKBP5–2 with gender in the prediction of EAC net of other main effects and control variables, the interaction effect was significant (*β* = −.113, *p* = .042). Probing simple slopes, controlling age, indicated a stronger association of demethylation at FKBP5–2 with EAC for men (*b* = −.202, *p* = .000) than for women (*b* = −.064, *p* = .138).

### Hypothesis 3: examination of indirect effects

We next examined whether the observed significant incubation effect resulted in additional significant indirect effects from early danger exposure to accelerated aging and/or accelerated cardiac risk through association with EAC. As shown in Table 3, indirect pathways estimated using bootstrapping with 30,000 resamples were significant for each of the outcomes, with significant effects on AccPCGrim, IE = .010 [.002, .024]; AccCardiac, IE = −.004, [−.011, −.001]; and AccPACE, IE = .002 [.000, .008]. In each case, all individual components of the pathway were also significant.

For completeness, in Table 3 we provide indirect effect estimates for behavioral and immune pathways, showing that these pathways remained significant even after the addition of the incubation pathway. Indirect effects from discrimination to health outcomes through the behavioral pathway were all significant. In addition, there was also a significant indirect effect to AccPCGrim through early self-reported smoking/drinking, IE = .030 [.013, .053]. Likewise, indirect effects from childhood exposure to danger to health through the inflammation pathway were all significant. Lastly, there were significant indirect effects from childhood exposure to danger to AccCardiac, IE = −.019 [−.040, −.005], and to AccPACE, IE = .014 [.002, .031] through FKPB5.

### Description of the resulting model

**Figure 2.**
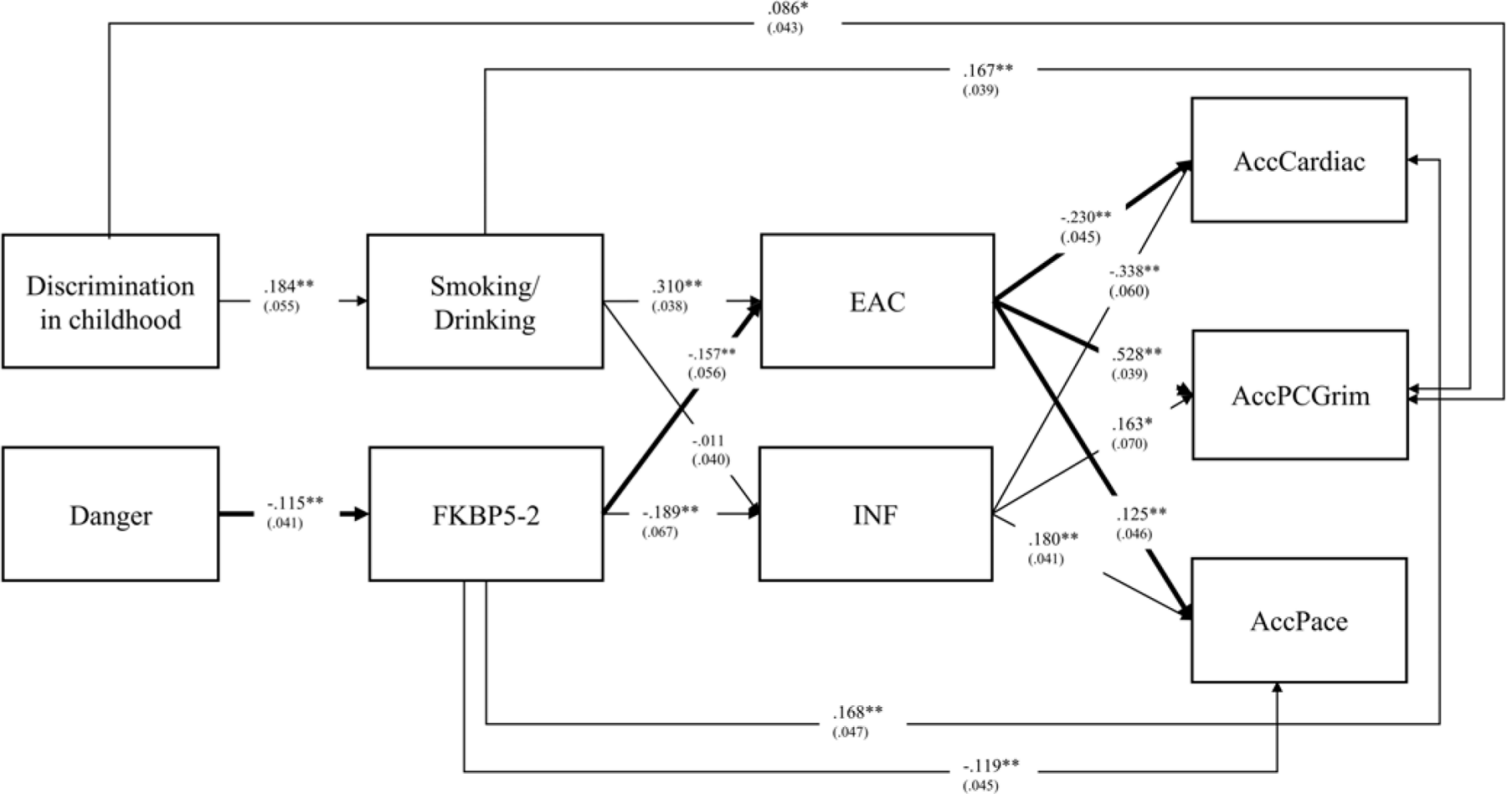
The dual pathway model linking childhood exposure to danger and discrimination at age 10 to accelerated epigenetic aging and cardiac risk via indirect effects through the substance use pathway leading to EAC and the inflammatory pathway leading to INF, with the addition of incubation effects on EAC. All pathways were freely estimated. Note: *N* = 449 (FIML), chi-square = 7.163, df = 4; p-value = 0.1275; RMSEA: =.042. CFI = .997; SRMR = .015. Gender controlled in all variables. Age controlled for DVs (AccCardiac; AccPCGrim; AccPace). Danger = exposure to dangerous environments in childhood; EAC = composite Non-self-report elevated alcohol consumption; INF = inflammatory to antiviral cell-type-ratio.

The full model that includes the hypothesized incubation pathway and controls for gender and age, is illustrated in Figure 2. All pathways were freely estimated. As can be seen, the model fits the data well: Chi-square = 7.163, df = 4, *p* = 0.1275; RMSEA = .042; CFI = .997; SRMR = .015. Consistent with the preceding analyses, the initial links in the incubation pathway from childhood danger exposure to FKBP5–2 (β = −.115, *p* = .005), and the pathway from FKBP5–2 to EAC (β = −.157, *p* = .005) were significant. However, the pathways from childhood danger exposure to early smoking/drinking and from Discrimination in Childhood to FKBP5 were not significant. Likewise, all pathways from EAC to young adult health outcomes were significant, with β = .528 for the pathway to AccPCGrim, β = .125 for the pathway to AccPACE, and β = −.230 for the pathway to AccCardiac.

### Sensitivity and exploratory analyses

**Figure 3.**
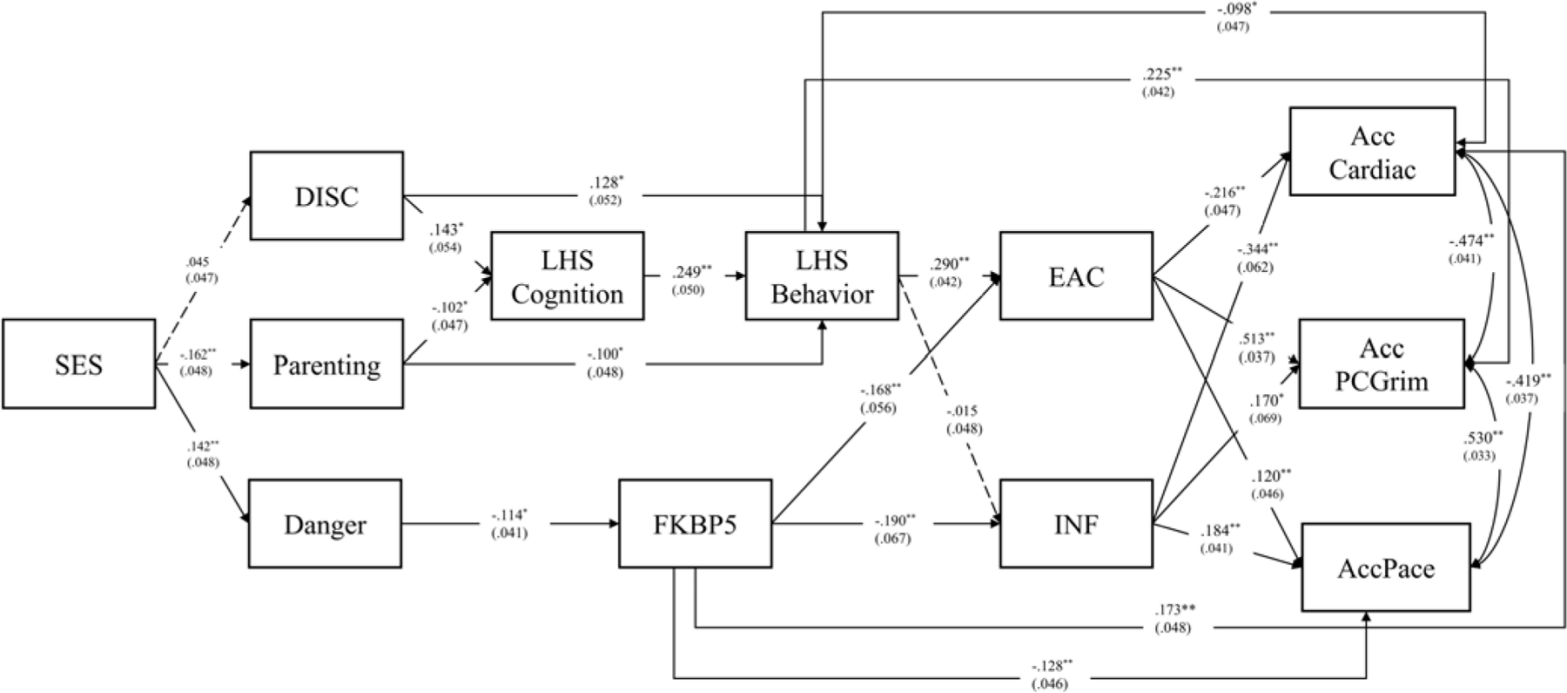
The dual pathway model linking childhood exposure to danger and discrimination at age 10 to accelerated epigenetic aging and cardiac risk with all variables from Beach et al. (2022a) included. All pathways were freely estimated. Note: the incubation pathway remained significant as were all pathways from EAC to outcomes. *N* = 449; chi-square = 10.356, df = 7. *p* = .1693; RMSEA = .033, CFI = .997, SRMR = .017. DISC = discrimination; danger = exposure to dangerous environments in childhood; EAC = composite Non-self-report elevated alcohol consumption; INF = inflammatory to antiviral cell-type-ratio.

To examine potential sensitivity of the model to excluded predictors from the original dual pathway model, we re-examined the model by including all predictor variables from the original model (Beach et al., 2022a). This expanded model included the addition of SES, parenting, and risk-taking cognition (LHS cognition), and used the original measure of risky behavior at the transition to adulthood (LHS behavior) that included marijuana use in addition to cigarette smoking and binge drinking. As can be seen, adding these variables did not change the pattern of significant associations (see Figure 3): the pathways from danger exposure to FKBP5–2 (β = −.114) and from FKBP5–2 to EAC (β = −.168) remained significant. Likewise, all pathways from EAC to outcomes remained significant. Accordingly, the additional predictors contributed to the model in expected ways, but the incubation effect remained significant.

**Figure 4:**
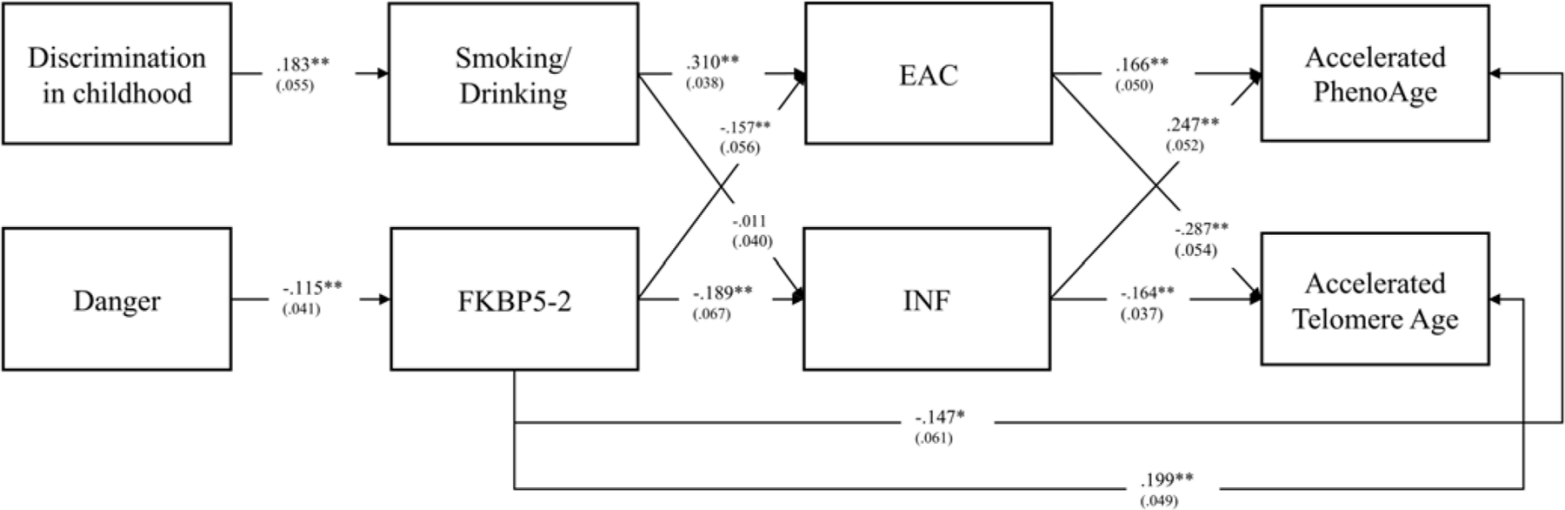
The Dual Pathway Model using alternative Accelerated DNAm aging indices (PhenoAge and Telomere Age) showing the same pattern of effects for incubation, behavioral, and inflammation pathways. All pathways were freely estimated. Note: N = 449; Chi-square = 7.067, df = 4; p-value= 0.1324; RMSEA: .041. CFI =.992; SRMR = .016. Danger = Exposure to Dangerous environments in childhood; EAC = Composite Non-Self-Report Elevated Alcohol Consumption; INF = Inflammatory to Antiviral Cell-Type-Ratio.

We also conducted sensitivity analyses to examine robustness with regard to use of alternative health-related DNAm indices of accelerated aging (see Figure 4). We used the two additional indices examined previously by Beach et al. (2022a). Accelerated PhenoAge (Levine et al., 2018) provides a useful marker of elevated risk for early onset morbidity and chronic illness (Horvath & Raj, 2018). Telomere length (Lu et al., 2019b) provides a DNA methylation-based index of telomere length. Using these two indices to assess accelerated aging, we found patterns of effects similar to those observed with AccPCGrim and DunedinePACE. All links in the incubation pathway remained significant as did those for the behavioral and inflammation pathways.

To further explicate cardiometabolic effects of EAC we also examined associations with self-reported diabetes at age 29 (see Supplementary Figure S1) and measured HbA1c at age 29 (see Supplementary Figure S2), showing significant effects of EAC on early onset of problems in these areas, with other associations in the model unchanged. INF was significantly associated with self-reported diabetes but not with HbA1c.

In addition, in Supplementary Figure S3 we show the model using self-reported binge drinking in young adulthood (age 29) rather than EAC, and again found a significant association of FKBP5–2 with young adult drinking net of the effect of drinking reported earlier at the transition to adulthood, supporting the presence of a delayed incubation effect from exposure to danger to elevated adult binge drinking. Self-reported binge drinking at age 29 was not significantly associated with accelerated aging or cardiac risk.

### Exploration of gender effects of EAC on aging outcomes

**Figure 5.**
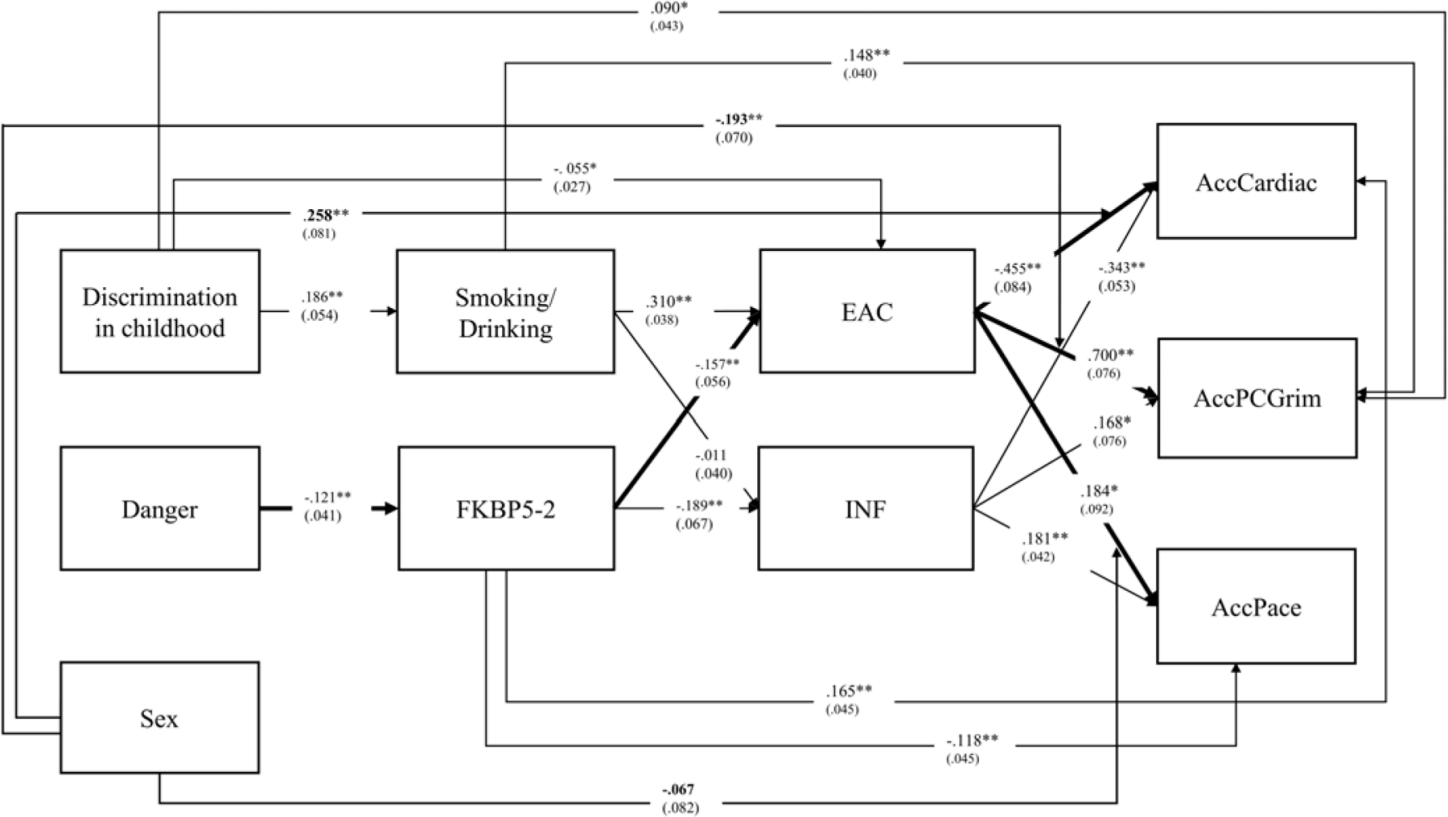
The dual pathway model linking childhood exposures to accelerated epigenetic aging outcomes and cardiac risk via indirect effects, with the addition of three interaction terms from SEX*EAC to outcomes to test sex differences. Note: *N* = 449; chi-square = 2.177, df = 4; *p*-value = 0.7032; RMSEA: .0000. CFI = 1.000; SRMR = .011. Danger = exposure to dangerous environments in childhood; EAC = composite Non-self-report elevated alcohol consumption; INF = inflammatory to antiviral cell-type-ratio.

To explore potential potential moderation of the associations between EAC and health outcomes by gender, we conducted analyses of the full dual pathway model introducing interaction terms between gender and EAC. As can be seen in Figure 5, modest but significant gender differences emerged for AccPCGrim (β = −.193, *p* = .005) and AccCadiac (β = .258, *p* = .001), but not for AccPace (NS). We explicated the significant interactions in Supplementary Figures S4 and S5. These showed that in both cases the impact of EAC on health outcomes was somewhat stronger for women than for men.

## Discussion

Black Americans are disproportionately exposed to acute and chronic stressors (Williams, 2018) and these stressors, particularly those occurring early in life, have the potential to promote vulnerability to elevated alcohol consumption across the lifespan (Nusslock & Miller, 2016). In the dual pathway model (Beach et al., 2022a) we hypothesized there would be equifinality with regard to the impact of different facets of adverse childhood experiences on accelerated aging. Specifically, although we hypothesized independent mechanisms of effect from different facets of adverse early exposures, we anticipated these pathways would converge to jointly predict negative health effects in young adulthood as measured by DNAm-based accelerated aging measures. In the current investigation, we added considerations from the NIN framework (Nusslock & Miller, 2016) suggesting that there might also be delayed cross-over effects from the inflammatory pathway to the behavioral pathway and these would be observable when examining associations that emerged later in development. Based on this line of reasoning we expanded the model to include non-self-report assessment of alcohol use later in young adulthood (age 29), anticipating that if there were cross-over incubation effects, we would see a correlation between the inflammation pathway and the behavioral pathway that emerged at this time, despite there not having been an association between the inflammation pathway and earlier smoking/drinking. We also hypothesized that by contributing to increased problematic alcohol consumption in young adulthood, the incubation effect would contribute to accelerated epigenetic aging and coronary heart disease, and that this would result in additional significant indirect pathway from early exposure to danger to outcomes via elevated alcohol consumption (EAC). By controlling the effect of early risky substance use and using a non-self-report index of young adult EAC, we probed these additional health effects attributable to the immune pathway via effects on EAC, and so tested the long-term outcomes of the incubation effects hypothesized by the NIN model.

Testing for the presence of an incubation effect required that we add a measure of EAC in young adulthood. Given prior work suggesting that self-reported drinking in young adulthood was problematic (Andersen et al., 2017; Beach et al., 2022b), we created an index comprising three non-self-report measures of EAC, allowing us to stringently test whether processes that appeared independent at the transition to adulthood (ages 18 to 24) might show cross-over associations when examined in young adulthood. Using a simplified theoretical model to focus attention on the hypothesized incubation effect, we replicated and extended the model presented by Beach et al. (2022a). Behavioral and inflammatory pathways continued to be significant predictors of health-related outcomes, including both DNAm-based accelerated aging measures and the newly added index of increased cardiac risk. At the same time, a significant, incubation-related pathway was observed, supporting expectations based on the NIN model. Sensitivity analyses demonstrated that the model was robust with regard to the addition of other theoretically relevant predictors (Gibbons et al., 2012) and also robust with regard to other DNAm-based accelerated aging outcomes trained on health-related outcomes, specifically PhenoAge (Levine et al., 2018) and telomere length (Lu et al., 2019b), as well as predicting early development of diabetes and elevated HbA1c. The results indicated that there were delayed patterns of association emerging in young adulthood by which early exposure to danger had significant indirect effects on health-related outcomes due to its indirect delayed association with heavy alcohol consumption in young adulthood, and these effects were in addition to inflammatory pathway associations.

The results support the hypothesis that early adverse exposures can contribute to elevated alcohol consumption in young adulthood through a process independent of early onset substance use, and by doing so can contribute to increased cardiac risk as well as general “weathering” (Geronimus et al., 2020) as indicated by health-related measures of DNAm-based measures of accelerated aging. These findings also support the perspective that the unfair and disproportionate weathering of Black men and women begins early in life and is multi-faceted (see also Brody et al., 2016, 2021; Geronimus, 1992), underscoring the importance of examining complex models of its effects. The pathways of inflammation and behavior examined in the current study exist within a context of structural racism that ensures that stressors proliferate and potentially become chronic once they are initiated. Likewise, stressors and stress reactions are often compounded with traumatic experiences across the lifespan (Carter et al., 2021). This context results in increased adverse consequences of alcohol use for Black men and women (Friedman et al., 1996; Zapolski et al., 2014), and reinforces pro-inflammatory contexts. As has been shown previously, SES also plays a role, at a minimum, in setting the stage for the behavioral and inflammatory pathways, and also provides a structural context that intensifies the impact of negative outcomes on health and well-being (O’Dell et al., 2021). This intensifying context may also partially account for prior observations that inflammation is associated with alcohol abuse for Black adults but not for White adults (Ransome et al., 2018), suggesting the importance of testing the incubation hypothesis for a range of marginalized groups.

It is possible that the incubation effect is more characteristic for Black youth, and particularly for Black males, relative to White youth given a greater tendency for abstinence in adolescence followed by increased adult-onset use and misuse (Banks & Zapolski, 2018; Finlay et al., 2012; Keyes et al., 2015; McCabe et al., 2007; Roberts et al., 2016). Likewise, incubation effects may be a particularly important process to address because patterns of drinking suggest decreased overall regular drinking but increased binge drinking among Black adults who drink in mid-adulthood (Pamplin et al., 2020).

One implication of the results linking exposures in childhood to adverse health outcomes in adulthood is the need to reduce the exposure of children to contexts that initiate cascades of problematic health behaviors and inflammation (Zapolski et al., 2014). This may take the form of action at the level of policy, or institutional change, and/or broader promotion of health equity, inclusion, and social justice. At the same time, the dual pathway model suggests downstream opportunities to protect and repair damaging processes that have already occurred. Efforts to reduce early exposures and to mitigate the impact of exposures that have already occurred may require attention to multiple key opportunities for intervention. In particular, the dual pathway model suggests that effects of discrimination on behavior may be apparent relatively early, but effects of the inflammation pathway may be less apparent until later in adulthood. Family-based interventions to protect against the adverse behavioral and immune effects of early exposures are available, with some having shown impact on DNAm-based measures of accelerated aging (Brody et al., 2016) as well as behavioral (Brody et al., 2010) and inflammatory (Miller et al., 2014) pathway effects. Accordingly, family-based interventions may help buffer impacts of early exposures on health via reduced stress effects or via increased parental influence on the behavioral and inflammatory pathways set in motion by childhood exposures. Delayed inflammation pathway effects (i.e., incubation effects) may require additional novel approaches to prevent escalation of alcohol or other substance use problems arising between the transition to adulthood and young adulthood. One possibility is to focus on the potential buffering role of newly emerging relationships in young adulthood that may begin to supplement relationships with parents (Beach et al., 2019) and that may begin to counter enduring vulnerabilities produced by childhood exposures (Simons et al., 2019).

Interestingly, exploratory analyses also indicated that there were significant sex differences in the strength of the incubation effect. In particular, we found modestly stronger effects for the incubation effect among Black men than among Black women. This is in keeping with prior research suggesting that chronic immune activation due to early exposures could influence neurocognitive architecture with implications for behavior (Nusslock & Miller, 2016), but that there is greater change in substance use between the transition to adulthood and young adulthood for men than for women (Chen et al., 2018). We also explored possible sex differences in effects on outcomes and found that there were significantly stronger associations between EAC and both AccPCGrim and Accelerated Cardiac Risk for Black women than for Black men. We did not observe significant sex differences in associations with AccPACE.

Despite the consistencies across sensitivity analyses, the specific mechanisms involved in linking childhood exposures to young adult health-related outcomes will require further explication. It would be useful, for example, to directly test indices of circulating peripheral cytokines both early in development and later in development to examine other ways of characterizing the immune pathway and so better capture the role of chronic inflammation. Accordingly, future research may expand this model further by better probing the inflammation – EAC relationship, particularly if it is possible to do so in the context of longitudinal research with early assessment of multiple facets of immune function and both early and later measures of alcohol use. Likewise, it would be useful to have earlier measures of change in level of FKBP5–2 as well as measures of change in DNAm-based measures of aging. In the current investigation, only one time point allowed for genome wide methylation assessment.

An additional limitation of the current manuscript is that the assessment of childhood danger was based on a relatively brief measure ascertained when youth were 10 years old. Future work using multiple sources of information and more in-depth assessments of awareness and impact of community violence may strengthen our understanding of children’s awareness of community danger and how this varies across different communities and racial/ethnic groups. In addition, although the longitudinal nature of this study is an important strength, extended follow-ups necessarily result in attrition. Our analyzed sample did not differ from the sample collected at baseline in terms of the two baseline predictors used in our model, but did differ in terms of age and sex. Accordingly, these demographic characteristics were controlled in all longitudinal models. However, it is possible that those included may have differed in other ways that we did not control.

Also of interest is the potential interaction with genetic risk and how this influences early (behavioral pathway) vs later (immune-related pathway) effects. Prior findings show that genetic influences in substance use are stronger later in life than during adolescence (e.g., Dick, 2011; Meyers & Dick, 2010) and may reflect somewhat different genetic risk processes. This prior work raises the possibility that genetic risk for externalizing behavior and genetic risk for adult alcohol dependence may interact with behavioral and immune-related pathways differently, with genetic risk for adult alcohol dependence amplifying delayed, immune-related pathway effects. This will be important to explore in future research. In addition, future research might examine whether early effects are driven, in part, by peer group selection effects. A stronger causal argument would also be possible if the dual pathway model were tested using intervention or quasi-experimental designs.

It also would have been useful had we been able to use non-self-report indicators of alcohol use at earlier ages as well as in young adulthood to show change across a single metric. However, some non-self-report assessments of alcohol use may be less sensitive in adolescence or across the transition to adulthood. The level of alcohol consumption that is required to result in sufficient biological perturbations assessed by the ATS, the CDT, or the methyldetectR is not well understood and may exceed typical consumption levels prior to early adulthood. At the same time, self-reported relative level of alcohol consumption during adolescence and the transition to adulthood may be more valid than reports at later ages when more extreme values may be systematically underreported (Beach et al., 2022b).

Collectively, the current findings suggest that prevention of health consequences of childhood adversity may ultimately require dual, or multiple, foci to counter the impact of different childhood experiences of adversity. In addition to increased efforts to provide children safe environments free of danger and exposure to discrimination, it may also be useful to identify modifiable resilience resources or “constructed sources of resilience” that can prevent incubation and/or also decrease early onset of substance use.

## Supplementary Material

1

The supplementary material for this article can be found at https://doi.org/10.1017/S0954579425000264.

## Figures and Tables

**Table 1.** Descriptive statistics and correlations among study variables

	1	2	3	4	5	6	7	8	9	10	11
1. Danger	—										
2. Discrimination	.303**	—									
3. FKBP5	−.117*	−.045	—								
4. Smoking/Drinking	.023	.167**	−.080^†^	—							
5. EAC	−.002	.000	−.203**	.335**	—						
6. INF	.006	−.012	−.201**	.015	.270**	—					
7. AccPCGrim	−.013	.084^†^	−.169**	.372**	.657**	.324**	—				
8. AccPACE	.007	.087^†^	−.146**	.086^†^	.132**	.189**	.446**	—			
9. AccCardiac	.020	−.049	.260**	−.141**	−.326**	−.406**	−.554**	−.504**	—		
10. Age	.030	.181**	−.074	.108*	.005	.033	.000	.000	.000	—	
11. Sex	.034	−.090^†^	−.123**	.098*	.260**	.151**	.271**	−.246**	.055	−.043	—
Mean	1.397	1.640	.635	.000	.005	.260	.000	.000	.000	29.152	.383
SD	.498	.539	.037	.810	.775	.319	3.536	.129	.039	.752	.486

*Note: N* = 449 Black adults; 172 men and 277 women. FKBP5 = FKBP5 methylation (FKBP5–2); EAC = Composite Non-Self-Report Elevated Alcohol Consumption; INF = Inflammatory to Antiviral Cell-Type-Ratio; AccPCGrim = Accelerated of PCGrimAge; AccPACE = Accelerated of DunedinPACE; AccCardiac = Accelerated demethylation of Composite Cardiac markers; SD = Standard Deviation.

† *p *< .10.

* *p *< .05.

** *p* < .01.

**Table 2.** Regression results predicting elevated alcohol consumption from smoking/drinking and FKBP5–2 controlling age and sex, and examining the moderating effect of sex (N = 449)

	Model 1	Model 2	Model 3
Smoking/drinking	.315**	.306**	.309**
FKBP5-2	—	−.155**	−.083
Age	−.019	−.030	−.034
Sex	.229**	.210**	.205**
FKBP5-2*sex	—	—	−.113*
R-square	.165	.188	.196
Change R-square	—	.023	.008

Note: Values are for β.

* *p *< .05.

** *p* < .01.

**Table 3.** Indirect effects of significant pathways in Figure 1 showing significant incubation pathway effects in addition to significant behavioral and inflammation pathway effects

Paths	Effect	95% CI
Incubation Effect		
Danger −> FKBP5 −> EAC −> AccPCGrim	.010*	[.002, .024]
Danger −> FKBP5 −> EAC −> AccCardiac	−.004*	[−.011, −.001]
Danger −> FKBP5 −> EAC −> AccPace	.002*	[.000, .008]
Discrimination −> Smoking/drinking −> EAC −> AccPCGrim	.030**	[.013, .053]
Discrimination −> Smoking/drinking-> EAC −> AccCardiac	−.013**	[−.027, −.006]
Discrimination −> Smoking/drinking −> EAC −> AccPace	.007*	[.002, .016]
Discrimination −> Smoking/drinking −> AccPCGrim	.031*	[.011, .062]
Inflammation Pathway Effects		
Danger −> FKBP5 −> INF −> AccPCGrim	.004*	[.000, .013]
Danger −> FKBP5 −> INF −> AccCardiac	−.007*	[−.016, −.002]
Danger −> FKBP5 −> INF −> AccPace	.004*	[.001, .011]
Danger −> FKBP5 −> AccCardiac	−.019*	[−.040, −.005]
Danger −> FKBP5 −> AccPace	.014*	[.002, .031]

Note: All significant indirect effects in Figure 1 from early exposure to outcomes of AccPCGrim, AccPace, and AccCardiac. The first set of effects are those tied to the incubation hypothesis and bolded in the heuristic model. The second set of effects are behavioral pathway effects from early exposure to discrimination to outcomes. The third set are inflammation pathway effects from early exposure to danger to outcomes (N = 449, FIML). All significant indirect pathways include an effect estimate and a 95% confidence interval [CI].

* *p* < .05.

** *p* < .01.
