# Supplementary material for: Childhood exposure to danger increases Black youths’ alcohol consumption, accelerated aging, and cardiac risk as young adults: A test of the incubation hypothesis
Source: Dev Psychopathol. Author manuscript; Available in PMC 2025 Oct 28. (PMC12353583; doi:10.1017/S0954579425000264)

**Supplementary Table S1.**

Comparisons of participants lost to attrition and included in analyses on baseline predictors (Danger and Discrimination) as well as Demographics (Age and Gender).

|  | Attrition  (n = 440) |  | Non-Attrition  (n = 449) |  |  |  |
| --- | --- | --- | --- | --- | --- | --- |
|  | Mean  (*SD*) |  | Mean  (*SD*) |  | *t-value* | *p-value* |
| Danger | 1.357  (.471) |  | 1.397  (.499) |  | 1.247 | .213 |
| Discrimination | 1.613  (.511) |  | 1.640  (.540) |  | 0.754 | .451 |
| Age | 10.620  (.629) |  | 10.510  (.641) |  | -2.642** | .008 |
| Sex | 0.540  (.499) |  | 0.380  (.487) |  | -4.843** | <.001 |

*Note*: Variables measured at baseline assessment.

Supplementary Table 2.  The inter correlation of the three non-self report measures and self-reported smoking/drinking at W4, W5, and W6

|  | 1 | 2 | 3 | 4 | 5 | 6 |
| --- | --- | --- | --- | --- | --- | --- |
| 1.ATS | — |  |  |  |  |  |
| 2.CDT7 | .378^**^ | — |  |  |  |  |
| 3.alcoholR | .369^**^ | .407^**^ | — |  |  |  |
| 4.Self-report smoking W4 | .235^**^ | .078 | .128^**^ | — |  |  |
| 5.Self-report smoking W5 | .311^**^ | .157^**^ | .170^**^ | .482^**^ | — |  |
| 6.Self-report smoking W6 | .343^**^ | .108^*^ | .173^**^ | .538^**^ | .595^**^ | — |
| 7.Self-report smoking W7 | .183^**^ | .121^*^ | .117^*^ | .391^**^ | .522^**^ | .505^**^ |
| 8.Self-report Drinking W4 | .106^*^ | .064 | .137^**^ | .376^**^ | .227^**^ | .197^**^ |
| 9.Self-report Drinking W5 | .165^**^ | .110^*^ | .194^**^ | .199^**^ | .308^**^ | .202^**^ |
| 10.Self-report Drinking W6 | .159^**^ | .125^*^ | .186^**^ | .116^*^ | .153^**^ | .205^**^ |
| 11.Self-report Drinking W7 | .015 | .107^*^ | .117^*^ | .066 | .136^**^ | .135^**^ |
|  |  |  |  |  |  |  |
| Mean | .778 | .942 | -12.296 | .681 | .964 | 1.031 |
| SD | 2.907 | .948 | .360 | 13.325 | 1.636 | 1.640 |

^*^ *p* < .05, ^**^ *p* < .01

Supplementary Table 2. (Continued)

|  | 7 | 8 | 9 | 10 | 11 |
| --- | --- | --- | --- | --- | --- |
| 7.Self-report smoking W7 | — |  |  |  |  |
| 8.Self-report Drinking W4 | .213^**^ | — |  |  |  |
| 9.Self-report Drinking W5 | .205^**^ | .362^**^ | — |  |  |
| 10.Self-report Drinking W6 | .167^**^ | .295^**^ | .480^**^ | — |  |
| 11.Self-report Drinking W7 | .155^**^ | .176^**^ | .345^**^ | .419^**^ | — |
|  |  |  |  |  |  |
| Mean | .672 | .617 | 1.328 | 1.420 | .829 |
| SD | 1.414 | 1.096 | 1.496 | 1.499 | 1.159 |

^*^ *p* < .05, ^**^ *p* < .01

Supplementary Figure S1.  EAC is associated with self-reported Diagnosis of Diabetes at age 29.


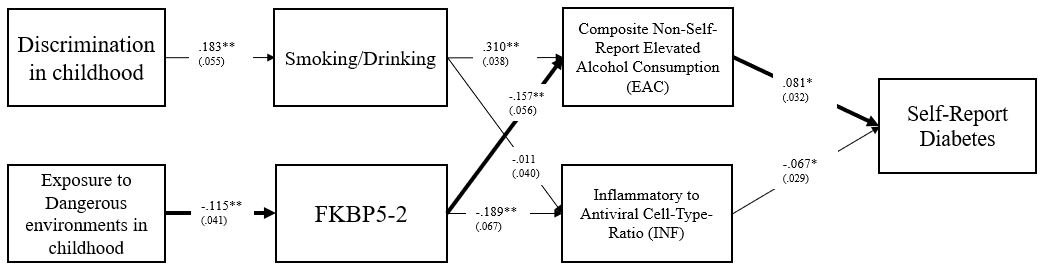


Note: Chi-square = 7.036, df = 4; p-value= 0.1340. RMSEA: .041. CFI =.981; SRMR =.017; N = 449 (FIML).

Supplementary Figure S2.  EAC is associated with measured levels of HbA1c at age 29.


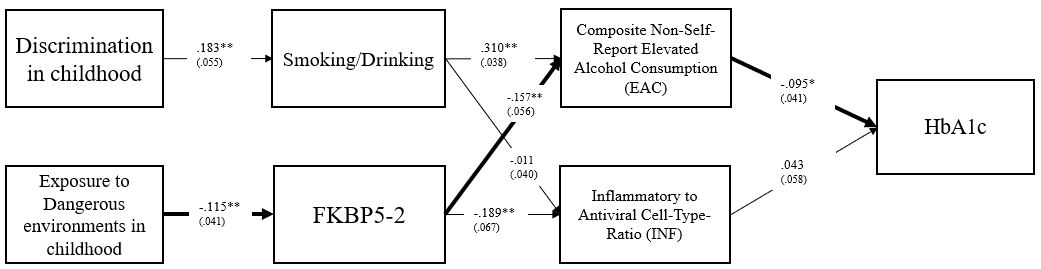


Note: Chi-square = 7.066, df = 4; p-value= 0.1324. RMSEA: .041. CFI =.981; SRMR =.017; N = 449 (FIML).

Supplementary Figure S3.  There is a significant incubation effect using self-reported Binge drinking in young adulthood (Age 29) in place of EAC.


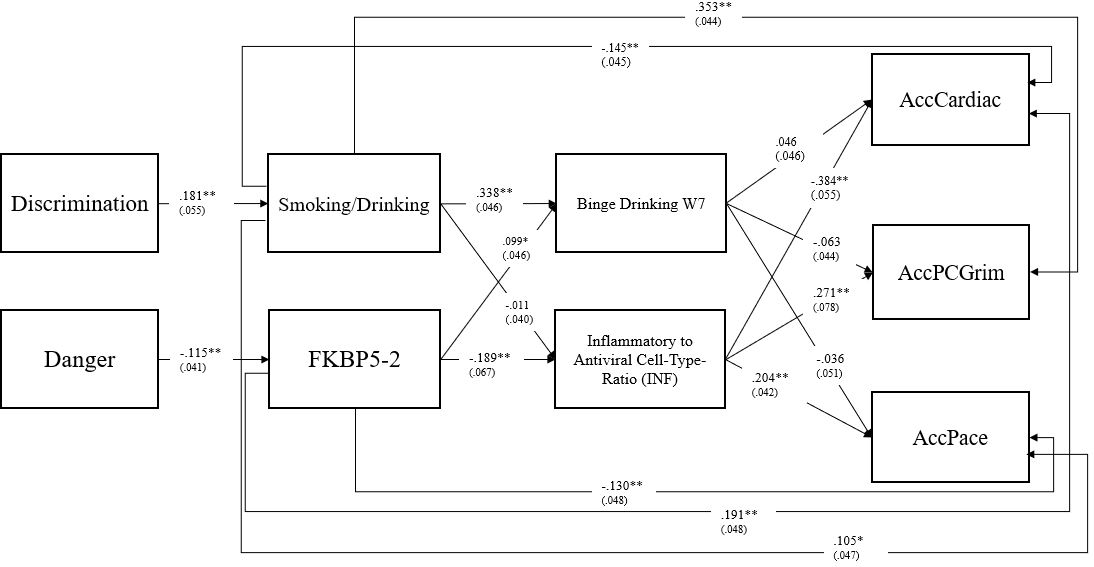


Note: Chi-square = 7.221, df = 4; p-value= 0. 1247; RMSEA: .042. CFI =.996; SRMR =.016; N = 449 (FIML).

Supplementary Figure S4. Effect of Elevated Alcohol Consumption on PCGrim Age by Gender.


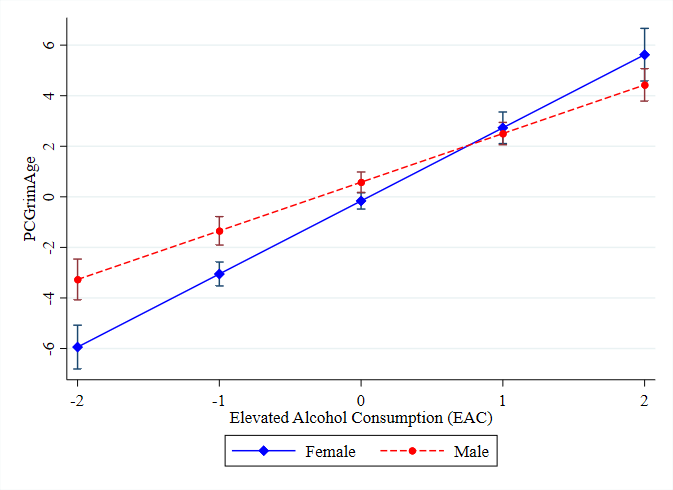


Supplementary Figure S5. Effect of Elevated Alcohol Consumption on Cardiac risk index by Gender.


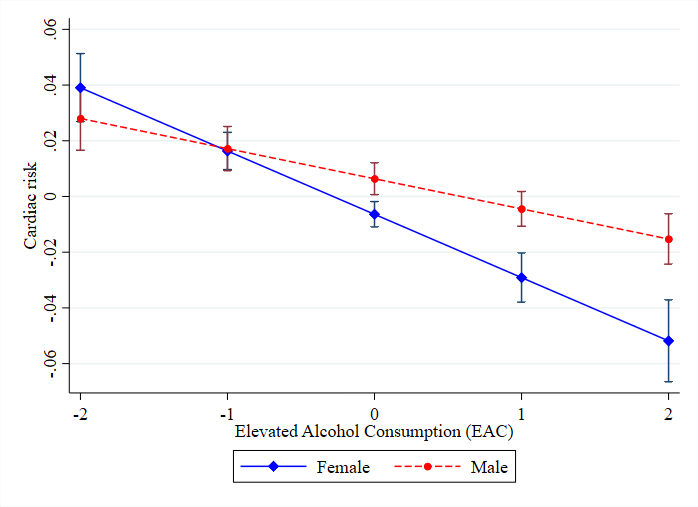

Supplement: 1 [file NIHMS2066433-supplement-1.docx]
